# The Complete Genome of *Brucella Suis* 019 Provides Insights on Cross-Species Infection

**DOI:** 10.3390/genes7020007

**Published:** 2016-01-26

**Authors:** Yuanzhi Wang, Zhen Wang, Xin Chen, Hui Zhang, Fei Guo, Ke Zhang, Hanping Feng, Wenyi Gu, Changxin Wu, Lei Ma, Tiansen Li, Chuangfu Chen, Shan Gao

**Affiliations:** 1 College of Medicine, Shihezi University, Xinjiang 832000, China; a201511281@126.com (Y.W.); d201511281@126.com (F.G.); 2 Co-Innovation Center for Zoonotic Infectious Diseases in the western region, Shihezi University, Xinjiang 832000, China; 3 College of Animal Science and Technology, Shihezi University, Xinjiang 832000, China; b201511281@126.com (Z.W.); zhanghui@shzu.edu.cn (H.Z.); e201511281@126.com (K.Z.); f201511281@126.com (H.F.); g201511281@126.com (W.G.); h201511281@126.com (C.W.); j201511281@126.com (T.L.); 4 College of Life Sciences, Nankai University, Tianjin 300071, China; c201511281@126.com; 5 College of Life Sciences, Shihezi University, Xinjiang 832000, China; i201511281@126.com

**Keywords:** Brucella, B. suis 019, complete genome, cross species, comparative genomics

## Abstract

*Brucella* species are the most important zoonotic pathogens worldwide and cause considerable harm to humans and animals. In this study, we presented the complete genome of *B. suis* 019 isolated from sheep (ovine) with epididymitis. *B. suis* 019 has a rough phenotype and can infect sheep, rhesus monkeys and possibly humans. The comparative genome analysis demonstrated that *B. suis* 019 is closest to the vaccine strain *B. suis* bv. 1 str. S2. Further analysis associated the rsh gene to the pathogenicity of *B. suis* 019, and the WbkA gene to the rough phenotype of *B. suis* 019. The 019 complete genome data was deposited in the GenBank database with ID PRJNA308608.

## 1. Introduction

*Brucella* is a genus of Gram-negative bacteria. They are small (0.5 to 0.7 by 0.6 to 1.5 µm), non-encapsulated, flagellated, facultatively intracellular coccobacilli [1]. *Brucella* causes the brucellosis in wild and domestic animals, even when transmitted from human to human. The brucellosis can have a considerable impact on human and animal health, as well as on economics, especially in developing countries where rural income relies largely on livestock breeding and dairy products [2]. The genus *Brucella* is generally classified into 10 species, which are *Brucella abortus*, *Brucella melitensis*, *Brucella suis*, *Brucella ovis*, *Brucella canis*, *Brucella neotomae*, *Brucella pinnipedialis, Brucella ceti, Brucella microti,* and *Brucella inopinata*, based on host preference and phenotypic characteristics [3,4]. Among these species, *B. melitensis*, *B. suis* and *B. canis* distribute more widely and virulently.

The strain named *B. suis* 019 infected sheep (ovine), rhesus monkeys and possibly humans. The 019 strain was first discovered in the 1980’s when the sheep epididymitis, usually caused by the *B. ovis*, broke out widely in the province of Xinjiang, China. After that, the 019 strain was isolated from the semen of sick sheep (ovine) and initially identified as a *B. ovis* strain by the serological and bacteriological tests [5]. Then, this identification was confirmed by the biochemical tests [6]. Later, the significant differences between the 019 strain and the other *B. ovis* strains were found through a series of experiments. The animal experiments proved the 019 strain infected rhesus monkeys and caused damage to many organs [7]. The molecular biological experiments showed some featured genes of the 019 strain were quite different from those of *B. ovis* [8]. In 2010, Wang *et al.* revealed that there were significant differences between the 019 strain and the 63/290 reference strain on both DNA and amino acid levels and concluded that the 019 strain was a unique local strain to Xinjiang [9]. However, the taxonomic status and infection mechanism of the 019 strain were still confusing.

In 2013, we assembled the draft genome of *B. suis* 019 using 90 bp Next-generation sequencing (NGS) technology and performed the comparative genomic analysis to reveal that the 019 strain belongs to *B. suis* and is far from *B. ovis* or *B. melitensis*. Although the *B. suis* 019 draft genome made effective progress, the draft genome missed some important information, e.g., genomic structure variation or rearrangement. Since pathogenic bacteria often exhibit a high degree of genomic rearrangement [10], we assembled the complete genome of *B. suis* 019 using the 250 bp NGS technology with Sanger sequencing confirmation. We also compared the *B. suis* 019 complete genome with the other 15 *Brucella* complete genomes to reach two research goals: 1) to confirm the taxonomic status of *B. suis* 019 strain based on the complete genome analysis; 2) to associate *B. suis* 019 strain’s rough phenotype and pathogenicity to some sequence features on the genome level.

## 2. Results and Discussion

### 2.1. Complete Genome Sequencing, Assembly and Annotation

The raw NGS data contained 2 × 688,568 paired reads with the length of 251 bp. After removing low quality regions, adapters and viral sequences, a total of 1,368,448 cleaned reads were produced for genome assembly. Using the cleaned reads, 14 and 6 scaffolds were assembled for chromosome 1 and 2. Then, we used the PCR plus Sanger sequencing to fill the gaps (Methods), producing the *B.* *suis* 019 complete genome (80× depth) containing two chromosomes with the length 2,098,391 bp and 1,204,433 bp, respectively (Supplementary file 1). The assembled *B.* *suis* 019 complete genome has a total sequence length of 3,302,824 bp, which is 3717 bp longer than the total length of the draft genome. This complete genome has the GC content 57.27%, which is very close to the GC content 57.28% of the draft genome. We predicted 1972 and 1119 proteins for 019 chromosome 1 and 2 (Supplementary file 2). Compared to the predicted 3529 ORFs using the draft genome, 3091 is closer to the total protein number of the other *Brucella* complete genomes (Table 1). All of the predicted proteins were annotated by the NCBI NR database and the Gene Ontology terms (Supplementary file 3). These proteins were predicted to involve 125 KEGG metabolism pathways (Supplementary file 4).

**Table 1:** 18 Brucella complete genomes.

Strain	Chr1_ID	Length	Gen#	Chr2_ID	Length	Gen#	CG%
* *B. abortus* A13334	NC_016795.1	2,123,773	2086	NC_016777.1	1,162,259	1102	57.40
*B. abortus* bv.1 str 9-941	NC_006932.1	2,124,241	2082	NC_006933.1	1,162,204	1103	57.22
*B. abortus* S19	NC_010742.1	2,122,487	2089	NC_010740.1	1,161,449	1106	57.22
*B. canis* ATCC 23365	NC_010103.1	2,105,969	2022	NC_010104.1	1,206,800	1131	57.24
*B. canis* HSKA 52141	NC_016778.1	2,107,023	2019	NC_016796.1	1,170,489	1098	57.24
*B. melitensis* 16M	NC_003317.1	2,117,144	2040	NC_003318.1	1,177,787	1107	57.22
*B. melitensis* ATCC 23457	NC_012441.1	2,125,701	2059	NC_012442.1	1,185,518	1117	57.22
*B. melitensis* biovar Abortus 2308	NC_007618.1	2,121,359	2086	NC_007624.1	1,156,948	1104	57.22
*B. melitensis* M28	NC_017244.1	2,126,133	2058	NC_017245.1	1,185,615	1118	57.22
*B. melitensis* M5-90	NC_017246.1	2,126,451	2062	NC_017247.1	1,185,778	1118	57.22
*B. melitensis* NI	NC_017248.1	2,117,717	2051	NC_017283.1	1,176,758	1112	57.23
*B. microti* CCM 4915	NC_013119.1	2,117,050	2024	NC_013118.1	1,220,319	1135	57.25
*B. ovis* ATCC 25840	NC_009505.1	2,111,370	2068	NC_009504.1	1,164,220	1122	57.19
*B. pinnipedialis* B2/94	NC_015857.1	2,138,342	2081	NC_015858.1	1,260,926	1188	57.20
*B. suis* 1330	NC_017251.1	2,107,783	2014	NC_017250.1	1,207,380	1130	57.25
* *B. suis* ATCC 23445	NC_010169.1	1,923,763	1848	NC_010167.1	1,400,844	1327	57.21
*B. suis* VBI22	NC_016797.1	2,108,637	2015	NC_016775.1	1,207,451	1132	57.25
*B. suis* 019	CP013963.1	2,098,391	1972	CP013964.1	1,204,433	1119	57.27

* These data were not used in this study. Chr1_len is the length of chromosome 1. Chr2_len is the length of chromosome 2. Gen# is the total gene number on this chromosome. Chr1_ID and Chr2_ID is the RefSeq or Genbank Accession Number.

### 2.2. Phylogenetic Analysis

**Figure 1:**
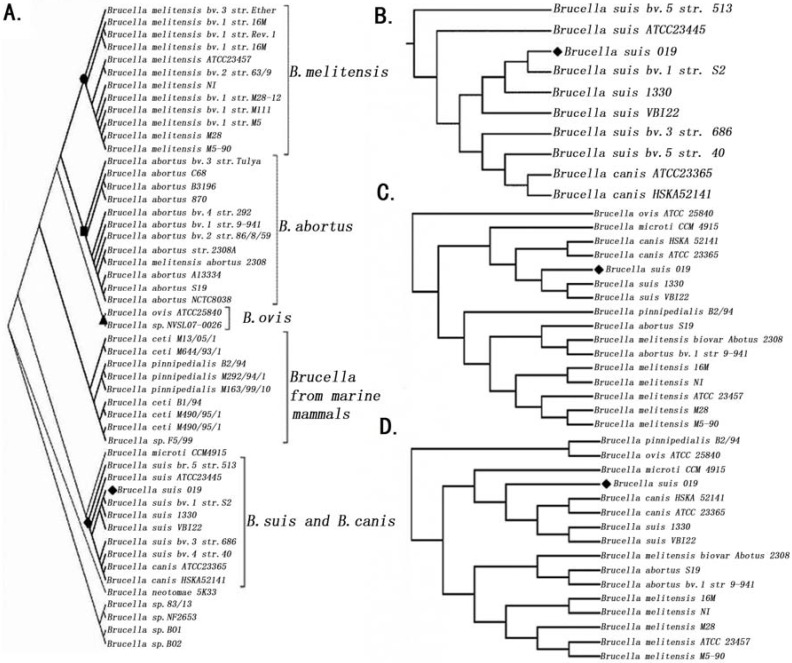
The phylogenetic trees. A. Phylogenetic Tree 1 was built using homologous genes from 51 *Brucella* genomes (including 019 draft genome). B. A magnified view of *Brucella suis* and *canis* clades was from Phylogenetic Tree 1. C. Phylogenetic Tree 2 was built using chromosome 1 sequences from 15 *Brucella* complete genomes (including 019 complete genome). D. Phylogenetic Tree 3 was built using chromosome 2 sequences from 15 *Brucella* complete genomes (including 019 complete genome).

Using 2,537 homologous genes from 51 *Brucella* genomes including the *B. suis* 019 draft genome (Methods), Phylogenetic Tree 1 was built to show six well-supported clades including *B. melitensis*, *B. abortus*, *B. ovis*, *Brucella* from marine mammals, *B. suis* and *canis*, and others (Figure 1A). These results confirmed that *B. suis* 019 belongs to the *B. suis* and is closest to *B. suis* bv. 1 str. S2 (Figure 1B). The vaccine strain *B. suis* S2, which had been developed in China in the 1970’s, was effective for oral vaccination of sheep, goats, cattle and pigs [11]. *B. suis* S2 has been widely used for prevention of animal brucellosis in China over many years.

Using chromosome 1 and 2 sequences from 16 *Brucella* complete genomes (Table 1), we built Phylogenetic Trees 2 and 3, separately (Figure 1C,D)*.* Phylogenetic Trees 2 and 3 had congruence with Phylogenetic Tree 1 on three points. The first point was that *B. suis* 019 belongs to *B. suis* species rather than *B. ovis.* The second point was that the debated *B. melitensis* *biovar Abotus* 2308 belongs to the *B. abortus* and is not a biovar of *B*. *melitensis*. The last point was that the *B. ovis* is far from other species, which confirmed a previous study. In that study, Foster *et al.* used 20,154 SNPs from 13 *Brucella* genomes to show that most *Brucella* species had diverged from their common *B. ovis* ancestor in the past 86,000 to 296,000 years, which preceded the domestication of their livestock hosts [12].

Using 16 complete genomes, we found the discrepancy between Phylogenetic Trees 2 and 3. Phylogenetic Tree 2 revealed a well supported topology that placed *B. ovis*, *B.* *microti*, *B. canis*, *B. suis*, *B. pinnipedialis*, *B. abortus* and *B. melitensis* in different clades (Figure 1C). Meanwhile Phylogenetic Tree 3 classified *B. ovis* and *B. pinnipedialis* into one clade and did not classify *B. suis* and *B. canis* into two well separated groups as Phylogenetic Tree 2 did. The latter phenomenon, named the paraphyly of *B. suis*, was discovered in two previous studies [12]. The results in these study suggested the paraphyly of *B. suis* could be attributed to chromosome 2 (Phylogenetic Tree 3). To further investigate the relationship between different *Brucella* species, we conducted a collinear analysis of 16 complete genomes to provide more detailed information on the genomic regions.

### 2.3. Comparative Genome Analysis Using 16 Complete Genomes

**Figure 2:**
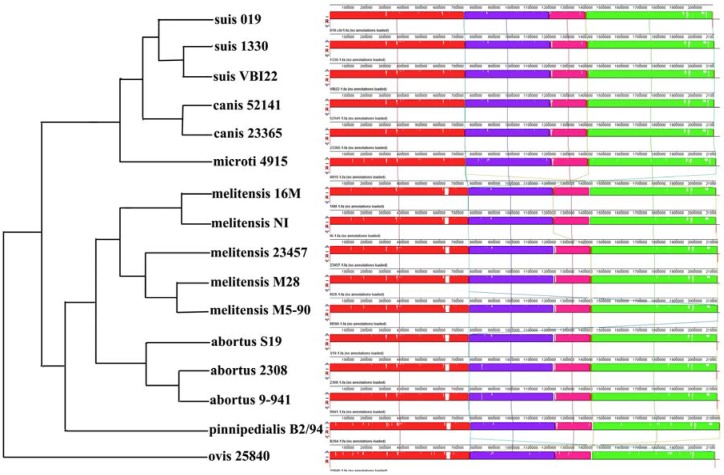
Nine LCBs on Brucella chromosome 1. Nine LCBs on chromosome 1 were built using 16 *Brucella* complete genomes. The blocks in the same color are connected by lines. A phylogenetic map of the strains derived from Phylogenetic Tree 2 (topology only) is on the left side.

**Figure 3:**
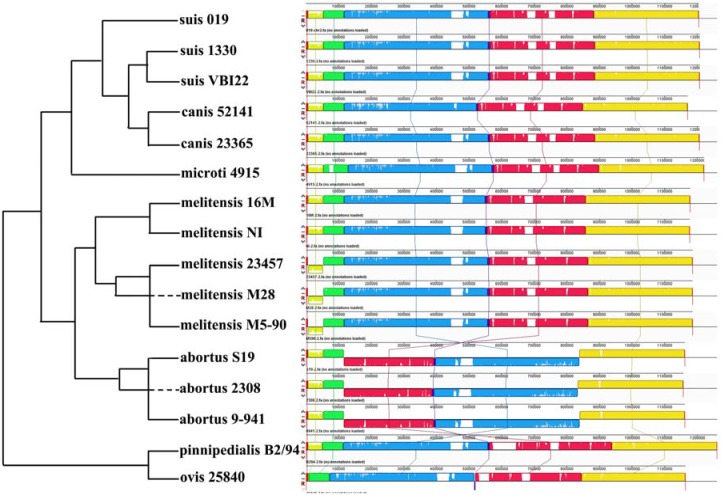
Seven LCBs on Brucella chromosome 2. Seven LCBs on chromosome 2 were built using 16 *Brucella* complete genomes. The blocks in the same color are connected by lines. A phylogenetic map of the strains derived from Phylogenetic Tree 3 (topology only) is on the left side.

Chromosome sequences from *B. suis* 019 and the other 15 *Brucella* species were aligned using the Mauve software (Methods). Mauve identified nine and seven locally collinear blocks (LCBs) for the 019 chromosome 1 and 2 (Supplementary file 5). LCBs are conserved segments that appear to be internally free from genome rearrangements [13]. Compared to the smaller LCB size in genomes of other genura (e.g., 78 LCBs in *Yersinia* [10] and 243 LCBs in *Shewanella* [14]), the large LCB size in *Brucella* genomes reflected the higher conservation in the *Brucella* genome structure. If not considering four LCBs with length 269, 300, 149 and 400 bp, five large LCBs with lengths 735,668, 465,416, 198,078, 6148 and 691,535 bp covered 99.63% (2,090,697/2,098,391) of the 019 chromosome 1 (Figure 2). Seven LCBs from the 019 chromosome 2 had the lengths 6490, 46,795, 63,955, 442,686, 3952, 319,146 and 321,153 bp (Figure 3). The total length of these seven LCBs was 1,204,177 bp, covering 99.98% (1,204,177/1,204,433) of the 019 chromosome 2. Overall, the *Brucella* chromosome 2 had a higher degree of genome rearrangements. A 46,795 bp LCB was almost absent on the *B*. *ovis* 25840 genome and inversed on the *B*. *melitensis* 23457, M28 and M5-90 genome. A 765,784 bp inversion including three LCBs was observed on the *B*. *abortus* S19, 2308 and 9-941 genome. A 3952 bp inversion was observed on the *B*. *ovis* 25840 genome. These results supported a previous conclusion that *Brucella* chromosome 2 is more dynamic, perhaps owing to its hypothesized origin as a plasmid [12].

**Figure 4:**
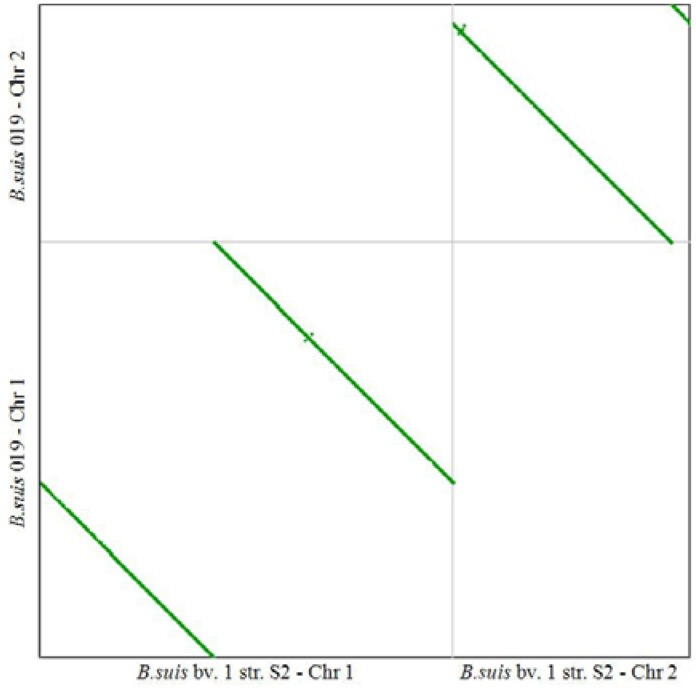
The syntenic map between the B. suis S2 and 019. **T**he syntenic map between the *B. suis* S2 and *B. suis* 019 strain was acquired on the CoGe website. The CDS sequences of *B. suis* S2 chromosome 1 and 2 used CP006961.1 and CP006962.1 from the GenBank database.

Since *B. suis* 019 is closest to *B. suis* S2 among all the *B. suis* species (Figure 1A), we conducted a CDS syntenic analysis between the *B. suis* 019 complete genome and the *B. suis* S2 draft genome. The results showed a good conservation of synteny and collinearity between *B. suis* 019 and the *B. suis* S2 genome (Figure 4). Then, we blasted all the CDS sequences of *B. suis* S2 to the *B. suis* 019 complete genome. All of the 3230 CDS sequences of *B. suis* S2 could be covered by the hits from the *B. suis* 019 genome, 99.54% (3215/3230) of which have the identity 100% to the query sequences. We also blasted all the CDS sequences of *B. suis* 019 to the *B. suis* S2 draft genome. All of the 3091 CDS sequences of *B. suis* 019 were able to be covered by the hits from the *B. suis* S2 genome, 99.78% (3084/3091) of which have the identity 100% to the query sequences. The comparison between *B. suis* 019 and the *B. suis* S2 showed there were eight genes absent in the *B. suis* 019 (Table 2) and 19 genes with significant mutation between *B. suis* S2 and *B. suis* 019 (Supplementary file 6). Among 19 genes, four genes have a single copy in the *B. suis* 019 complete genome (Table 2). Particularly, we found that a 21 bp nucleotide deletion in the rsh gene resulted in a seven amino acid deletion QKRASGD. Based on search results from the NCBI website, we reported this as a new mutation of rsh gene.

The product of rsh named GTP pyrophosphokinase rsh (EC: 2.7.6.5) is a mediator of the stringent response that coordinates a variety of cellular activities in response to changes in nutritional abundance. Rsh is required for *Brucella* to express the type IV secretion system VirB, a major virulence factor of *Brucella* and therefore plays a role in adaptation to low-nutrient environments. This was evidenced using the Rsh deletion mutants in *B. suis* and *B. melitensis* in a previous study [15]. Comparative transcriptional analysis between *B. suis* 1330 wild-type and Δ*rsh* mutant showed the Rsh-dependent up-regulation of 198 genes and down-regulation of 181 genes, which together account for 11.6% of the genome [16]. The Rsh protein (Uniprot: Q8CY42) with a length of 750 AA (amino acids) has four Pfam domains, HD_4 (residues 26-176), RelA_SpoT (residues 235-346), TGS (residues 392-451) and ACT_4 (residues 669-747). The seven amino acid deletion (residues 37-43) belongs to the HD_4 domain. We used the PredictProtein server (https://www.predictprotein.org) to analyze the rsh properties and predicted that all of the deleted seven amino acids had the coil secondary structures. These seven amino acids were predicted in the disorder regions. Moreover, the Glutamine (Q) on residue 37 was predicted as a protein binding region. Compared to the other three single copy genes (Table 2), the rsh gene with the seven amino acid deletion is more likely to be associated to the acquired pathogenicity of the *B. suis* 019.

**Table 2:** Significant different genes between B. suis 019 and S2.

Gene-ID	Chr	Length	Copy	Product
BSS2_I0512	1	795	1	integrase catalytic subunit
BSS2_I0517	1	1119	>1	mannosyltransferase
BSS2_I0518	1	369	1	transposase
BSS2_I0519	1	342	1	IS5 family transposase orfB
BSS2_I0898	1	165	>1	hypothetical protein
BSS2_I1794	1	1929	1	hypothetical protein
BSS2_I1795	1	675	1	hypothetical protein
BSS2_II0527	2	1965	>1	cell wall surface protein
chr1_239	1	2232	1	gtp pyrophosphokinase rsh
chr1_277	1	1140	1	cytochrome c-type biogenesis protein
chr1_995	1	4782	1	outer membrane autotransporter barrel domain-containing protein
chr1_1847	1	777	1	3-mercaptopyruvate sulfurtransferase

Gene-ID has two formats. The format “BSS2_xxxx” is the tag name in the GenBank data CP006961.1 (*B. suis* S2 chromosome 1) and CP006962.1 (*B. suis* S2 chromosome 2). The format “chrx_xxxx” is the gene ID of *B. suis* 019 complete genome (Supplementary files 1 and 2). The first eight genes are absent in the *B. suis* 019 complete genome. The other four genes have significant mutation from *B. suis* S2 to *B. suis* 019.

### 2.4. Beta-Ketoadipate Pathway and Lipopolysaccharide

One important characteristic of *Brucella* is that it shares two pathways with the soil microorganisms. These two pathways, beta-ketoadipate pathway and homoprotocatechuate pathway are widely distributed among diverse soil microorganisms and play a central role in the processing and degradation of plant-derived aromatic compounds. The *B. suis* 1330 genome (GenBank: AE014291.4 and AE014292.2) includes these two intact pathways, the genes of which are located on chromosome 2 [17]. The homoprotocatechuate pathway includes eight protein-coding genes (AE014292.2: BRA1155–BRA1162). These genes numbered from chr2_1076 to chr2_1084 (Table 3) were found in *B. suis* 019 in the same order on chromosome 2 with sequence identity 100%. The beta-ketoadipate pathway includes 12 protein-coding genes (AE014292.2:BRA0636–BRA0647). One previous study showed that at least 1 of the 12 genes carried by every *Brucella* genome except *B. suis* 1330 has become a pseudogene and 12 genes are completely missing in *B. suis* ATCC 23445 [18]. These 12 genes numbered from chr2_443 to chr2_454 (Table 3) were found in *B. suis* 019 in the same order on chromosome 2 with sequence identity 100%.

**Table 3:** Featured genes of Brucella.

1330-ID	Chr	Start	End	019-ID	Start	End	Product
BRA0636	2	617674	618876	chr2_454	490638	491840	beta-ketoadipyl CoA thiolase
BRA0637	2	618885	619574	chr2_453	489940	490629	3-oxoadipate CoA-transferase, beta subunit
BRA0638	2	619571	620278	chr2_452	489236	489943	3-oxoadipate CoA-transferase, alpha subunit
BRA0639	2	620462	621232	chr2_451	488282	489052	transcriptional regulator PcaR, putative
BRA0640	2	621239	622156	chr2_450	487358	488275	pobR protein
BRA0641	2	622269	623438	chr2_449	486076	487245	p-hydroxybenzoate hydroxylase
BRA0642	2	623848	624756	chr2_448	484758	485666	transcriptional regulator PcaQ
BRA0643	2	624850	625653	chr2_447	483861	484664	3-oxoadipate enol-lactone hydrolase
BRA0644	2	625657	626073	chr2_446	483441	483860	4-carboxymuconolactone decarboxylase
BRA0645	2	626070	626810	chr2_445	482704	483444	protocatechuate 3,4-dioxygenase, beta subunit
BRA0646	2	626812	627429	chr2_444	482085	482702	protocatechuate 3,4-dioxygenase, alpha subunit
BRA0647	2	627433	628497	chr2_443	481017	482081	3-carboxy-cis,cis-muconate cycloisomerase, putative
BRA1155	2	1155255	1156661	chr2_1084	1157262	1158668	aldehyde dehydrogenase family protein
BRA1156	2	1156818	1157627	chr2_1082	1156296	1157105	2,4-dihydroxyhept-2-ene-1,7-dioic acid aldolase
BRA1157	2	1157694	1157804	chr2_1081x	1156119	1156229	hypothetical protein
BRA1158	2	1157845	1158648	chr2_1081	1155275	1156078	2-keto-4-pentenoate hydratase
BRA1159	2	1158652	1159518	chr2_1080	1154405	1155271	fumarylacetoacetate hydrolase family protein
BRA1160	2	1159587	1160567	chr2_1078	1153356	1154336	catechol 2,3-dioxygenase (pseudo)
BRA1161	2	1160622	1162136	chr2_1077	1151787	1153301	5-carboxy-2-hydroxymuconate semialdehyde dehydrogenase
BRA1162	2	1162206	1162598	chr2_1076	1151325	1151717	5-carboxymethyl-2-hydroxymuconate delta isomerase
BR0058	1	64824	66455	chr1_179	820216	821847	phosphoglucomutase
BR0510	1	509856	511724	chr1_359	374961	376829	epimerase/dehydratase, putative
BR0511	1	511711	512718	chr1_358	373967	374974	glycosyl transferase, group 4 family protein
BR0517 *	1	516587	517366	chr1_353	369319	370098	formyltransferase, putative
BR0519 *	1	518244	519002	chr1_351	367683	368441	O-antigen export system ATP-binding protein RfbE
BR0520	1	518999	519781	chr1_350	366904	367686	O-antigen export system permease protein RfbD
BR0521	1	519796	520899	chr1_349	365786	366889	perosamine synthase, putative
BR0522	1	520907	521995	chr1_348	364690	365778	GDP-mannose 4,6-dehydratase
BR0529	1	525257	526375	NA	498887	498927	mannosyltransferase, putative
BR0537	1	529702	531039	chr1_345	361179	362516	phosphomannomutase, putative
BR0538 *	1	531064	532488	chr1_344	359730	361154	mannose-1-phosphate guanylyltransferase/mannose-6-phosphate isomerase
BR0539 *	1	532521	533693	chr1_343	358525	359697	mannose-6-phosphate isomerase
BR0540	1	533776	534885	chr1_342	357333	358442	glycosyl transferase, group 1 family protein
BR0615	1	606884	608995	chr1_269	283223	285334	membrane protein, putative
BR0981	1	948161	949393	chr1_1915	2041237	2042469	glycosyl transferase WboA
BR0982	1	949390	950949	chr1_1914	2039681	2041240	glycosyl transferase, group 1 family protein
BR1503 *	1	1457802	1458866	chr1_1441	1531664	1532728	lipopolysaccharide core biosynthesis mannosyltransferase LpcC
BRA0347 *	2	326808	328223	chr2_723	778336	779751	mannose-1-phosphate guanylyltransferase/mannose-6-phosphate isomerase
BRA0348	2	328220	329653	chr2_722	776906	778339	phosphoglucomutase, putative

* Three groups of featured genes are separated by a blank line. The first group of 12 genes involves in the beta-ketoadipate pathway. The second group of 8 genes involves in the homoprotocatechuate pathway. The third group of 19 genes are indicated as being important in producing smoothness. 1330-ID uses tags in the GenBank data AE014291.4 and AE014292.2. 019-ID uses gene ID of *B. suis* 019 genome (Supplementary files 1 and 2).

Lipopolysaccharide (LPS) is the major structural component of the outer membrane of gram-negative bacteria. It is composed of a lipid core, a core oligosaccharide, and a distal *O*-polysaccharide (*O*-PS) side chain [19]. The presence of the intact *O*-PS produces smooth phenotypes in *B. melitensis, B. suis* and *B. abortus,* while the absence or disruption of *O*-PS produces rough phenotypes in *B. canis* and *B. ovis* with the lipid core and the core oligosaccharide. An unexpected finding on the *B. suis* 019 was its rough morphology. Several studies indicated specific genes are important for the development of the smooth phenotype in *Brucella* [19]. Until now, 19 genes have been indicated as being important in producing smoothness. The disruption of 13 genes, Pgm (BR0058), WbkD (BR0510), WbkF (BR0511), Wzm (BR0520), Per (BR0521), Gmd (BR0522), WbkA (BR0529), ManB (BR0537), WbkE (BR0540), Wa** (BR0615), WboA (BR0981), WboB (BR0982) and ManBcore (BRA0348), resulted in a rough phenotype in *B.* *melitensis* and six genes, WbkC (BR0517), Wzt (BR0519), ManC (BR0538), ManA (BR0539), LpcC (BR1503) and ManCcore (BRA0347), were identified as playing roles that had not been fully determined (Table 3). Based on the alignment results, we found that the WbkA gene was disrupted in the *B. suis* 019 complete genome with the other 18 genes 100% identical to their orthologs in *B. suis* 1330. Previous studies have demonstrated that spontaneous excision of the WbkA glycosyltranferase gene was a cause of dissociation of smooth to rough *Brucella* [20]. Therefore, we proposed that the disruption of the WbkA gene resulted in the rough *B. suis* 019.

## 3. Materials and Methods

### 3.1. Sample Preparation, DNA-seq Library Construction

The *B.* *suis* 019 strain was obtained from the key laboratory of prevention and control of animal disease, Shihezi University. This bacteria had been originally isolated from the sperm of sheep in the province of Xinjiang in China in 1983, then processed by freeze-drying for long-term preservation. In this study, *B.* *s*uis 019 was cultured at 37 °C for 72 h using the streak plate method. Single bacterial colonies were inoculated in the TS broth at 37 °C with shaking for 48 h. Bacteria were collected by centrifugation at 10,000 rpm for 1 min and washed twice with sterile deionized water. Total genomic DNA was extracted and purified by the GENEray™ Bacteria Whole Genome DNA Extraction Kit GK1072 (Generay Biotech, Shanghai, China). The DNA purity and concentration was measured by a NanoDrop™ spectrophotometer. DNA fragmentation was conducted using an ultrasound machine. DNA fragments of around 500 bp size were separated and collected using Agarose Gel Electrophoresis. Finally, one DNA library was constructed using the Illumina TruSeq™ (Illumina, San Diego, CA, USA) DNA Sample Prep Kits for the draft genome sequencing. The same procedure was conducted to construct another DNA library for the complete genome sequencing by a different experimenter one year later.

### 3.2. Draft Genome Sequencing, Assembly and Annotation

The DNA-seq library was sequenced using the Illumina HiSeq™ 2000 platform. *De novo* assembly of the *B.* *suis* 019 draft genome was performed using the SOAPdenovo 1.05 [21]. Gene prediction was performed using the software Glimmer 3.02 [22]. The raw NGS data contained paired reads with the left read length of 90 bp and right read length of 70 bp. We produced a total of 330 M bp cleaned NGS data, roughly covering 100 fold (100×) of the *B.* *suis* 019 draft genome. The assembled *B.* *suis* 019 draft genome has a total sequence length of 3,299,107 bp with the GC content 57.28%. This assembly produced 30 scaffolds and 722 contigs (Methods). The scaffold N50 and contig N50 is 259,978 bp and 7677 bp, respectively. We predicted 3,529 ORFs with the average length of 804 bp. This data was submitted to the GenBank WGS database with ID ANOZ00000000.

### 3.3. Complete Genome Sequencing, Assembly and Annotation

The DNA-seq library was sequenced using the Illumina HiSeq™ 2000 platform. After removing low quality regions, adapters and viral sequences, the cleaned reads were produced for genome assembly using the software Fastq_clean [23]. *De novo* assembly of the *B.* *suis* 019 genome was performed using the Celera Assembler version 8.1 [24] to produce scaffolds with the default setting. Blastn [25] searching was conducted against the NCBI bacterial genome database with the scaffolds to find the best matched genome *B. ceti* TE28753-12 as the reference genome. Based on the reference sequence NC_022907.1 and NC_022908.1, we applied the LASTZ and Chain/Net to order the scaffolds on two chromosomes, respectively. The gaps within and between the scaffolds were closed with the GapFiller [26]. Gene prediction was performed using the software Prodigal 2.60 [27]. All putative genes were annotated based on the NCBI NR database. Functional categorization by Gene Ontology (GO) terms and KEGG pathway annotation was carried out based on the best 20 blastx hits from the NR database using the Blast2GO software [24].

### 3.4. Phylogenetic Analysis Using Homologous Genes

Using all the annotated genes in the *B.* *suis* 1330 genome as a reference, we aligned the genes of the other 50 genomes to the reference genes using the blastn software. Taking the sequence identity 70% as threshold, we obtained a total of 2537 homologous genes from the alignment results. These homologous genes were linked into 51 super homologous sequences. The length of the super homologous sequences is about 2,226,048 bp covering more than 2/3 of the *Brucella* genome. The multiple alignment of 51 super homologous sequences was implemented using ClustalW 2.0 [28]. At last, a phylogenetic tree was built using the UPGMA (unweighted pair-group method with arithmetic means) method in the software MEGA 5.0 [29].

### 3.5. Comparative Genome Analysis Using 16 Complete Genomes

Removing two genomes due to their abnormal genome size (ATCC 23445) or GC content (A13334), we used *B.* *suis* 019 with 15 other complete genomes for the analysis (Table 1). The phylogenetic trees for chromosomes 1 and 2 were constructed using the software Mauve 2.4.0 with the default setting [13]. The collinear analysis and result display was also conducted using Mauve 2.4.0. To clearly display the collinear analysis result in Mauve, we rotated 15 genome sequences to roughly align the collinear regions to start from similar regions on the chromosome.

The syntenic analysis between *B.* *suis* 019 and *B.* *suis* S2 (v1, id25770) was conducted using the SynMap tool with default setting on the CoGe website. To be compatible with previous studies, the comparison between *B.* *suis* 019 and *B.* *suis* S2 used the GenBank data CP006961.1 (*B.* *suis* S2 chromosome 1) and CP006962.1 (*B.* *suis* S2 chromosome 2) (Table 2). The comparison between *B.* *suis* 019 and *B.* *suis* 1330 used the GenBank data AE014291.4 (*B.* *suis* 1330 chromosome 1) and AE014292.2 (*B.* *suis* 1330 chromosome 2) (Table 3).

## 4. Conclusions

In this study, we presented the complete genome of *B.* *suis* 019 and conducted comparative genome analysis. *B. suis* 019 was identified to be closest to the vaccine strain *B. suis* bv. 1 str. S2. Based on further analysis results, we associated the rsh gene to the pathogenicity of *B. suis* 019, and the WbkA gene to the rough phenotype of *B. suis* 019.

## Supplementary Materials

The following are available online at www.mdpi.com/link.

## Abbreviations

The following abbreviations are used in this manuscript:

### Abbreviations

NGS: Next-generation sequencing
LCBs: Locally Collinear Blocks
LPS: Lipopolysaccharide
*O*-PS: *O*-polysaccharide
GO: Gene Ontology
UPGMA: unweighted pair-group method with arithmetic means
