# Supplementary material for: The Complete Genome of Brucella Suis 019 Provides Insights on Cross-Species Infection
Source: Genes (Basel). 2016 Jan 26;7(2):7. doi: 10.3390/genes7020007 (PMC4773751; doi:10.3390/genes7020007)
Supplement: Supplementary file 1 [file genes-07-00007-s001.zip › suppl/supplementary_file_6.pdf]

| Query_name | Length | Single-copy | Function |
|---|---|---|---|
| brucella-chr1_1290 | 393 | ○ | transposase |
| brucella-chr1_1291 | 369 | ○ | transposase |
| brucella-chr1_1340 | 129 | ○ | hypothetical protein, partial |
| brucella-chr1_1847 | 777 | ● | 3-mercaptopyruvate sulfurtransferase |
| brucella-chr1_1918 | 369 | ○ | transposase |
| brucella-chr1_1919 | 609 | ○ | queuine trna-ribosyltransferas |
| brucella-chr1_1972 | 369 | ○ | transposase |
| brucella-chr1_239 | 2232 | ● | gtp pyrophosphokinase rsh |
| brucella-chr1_277 | 1140 | ● | cytochrome c-type biogenesis protein |
| brucella-chr1_347 | 417 | ○ | transposase orfb |
| brucella-chr1_705 | 393 | ○ | transposase |
| brucella-chr1_706 | 369 | ○ | transposase |
| brucella-chr1_995 | 4782 | ● | outer membrane autotransporter barrel domain-containing protein |
| brucella-chr2_1 | 255 | ○ | protein |
| brucella-chr2_2 | 882 | ○ | is3 family transposase orfb |
| brucella-chr2_256 | 393 | ○ | protein |
| brucella-chr2_257 | 369 | ○ | transposase |
| brucella-chr2_47 | 909 | ○ | is3 family transposase orfb |
| brucella-chr2_524 | 363 | ○ | transposase orfa |

**Query= brucella-chr1_239 # 247920 # 250151 # -1 # ID=1_239;partial=00;start_type=ATG;rbs_motif=GGAG/GAGG;rbs_spacer=5-10bp;gc_cont=0.577**
**(2232 letters)**

```
                                                                 Score    E
Sequences producing significant alignments:                      (bits) Value

gb|CP006961.1|                                                      3157   0.0

>gb|CP006961.1|
          Length = 2107842

 Score = 3157 bits (2209), Expect = 0.0
 Identities = 2232/2253 (99%), Gaps = 21/2253 (0%)
 Strand = Plus / Plus

Query: 1      atgatgcgccaatatgagcttgtggagcgtgtgcagcgatacaagcctgatgtgaacgag 60
              ||||||||||||||||||||||||||||||||||||||||||||||||||||||||||||
Sbjct: 642046 atgatgcgccaatatgagcttgtggagcgtgtgcagcgatacaagcctgatgtgaacgag 642105


Query: 61     gcgcttcttaacaaggcatatgtttatgccatgcagaaacacggcagtc----------- 109
              |||||||||||||||||||||||||||||||||||||||||||||||||
Sbjct: 642106 gcgcttcttaacaaggcatatgtttatgccatgcagaaacacggcagtcagaagcgggct 642165


Query: 110    ----------cctatttctcccatccgctggaagtggcggctattctcacagatatgcat 159
                        ||||||||||||||||||||||||||||||||||||||||||||||||||
Sbjct: 642166 tccggcgatccctatttctcccatccgctggaagtggcggctattctcacagatatgcat 642225


Query: 160    ttggacgaggcgacaatcgccatcgcgcttctgcacgacacgatcgaggataccacggcc 219
              ||||||||||||||||||||||||||||||||||||||||||||||||||||||||||||
Sbjct: 642226 ttggacgaggcgacaatcgccatcgcgcttctgcacgacacgatcgaggataccacggcc 642285


Query: 220    acccggcaggaaatcgaccagcttttcgggccggaaatcggcaagcttgtcgaggggctg 279
              ||||||||||||||||||||||||||||||||||||||||||||||||||||||||||||
Sbjct: 642286 acccggcaggaaatcgaccagcttttcgggccggaaatcggcaagcttgtcgaggggctg 642345


Query: 280    accaagctcaagaaactcgatctcgtttccaagaaggctgtccaggcggaaaacctgcgt 339
              ||||||||||||||||||||||||||||||||||||||||||||||||||||||||||||
Sbjct: 642346 accaagctcaagaaactcgatctcgtttccaagaaggctgtccaggcggaaaacctgcgt 642405
```

## The latter part of this record is omitted

**Query= brucella-chr1_239**
**(2232 letters)**
**Database: BSS2_I0634.fa**
**1 sequences; 749 total letters**

```
                                                                 Score    E
Sequences producing significant alignments:                      (bits)  Value

BSS2_I0634                                                          1461   0.0

>BSS2_I0634
          Length = 749

 Score = 1461 bits (3781), Expect = 0.0
 Identities = 742/749 (99%), Positives = 742/749 (99%), Gaps = 7/749 (0%)
 Frame = +1

Query: 1    MMRQYELVERVQRYKPDVNEALLNKAYVYAMQKHGS-------PYFSHPLEVAAILTDMH 159
            MMRQYELVERVQRYKPDVNEALLNKAYVYAMQKHGS       PYFSHPLEVAAILTDMH
Sbjct: 1    MMRQYELVERVQRYKPDVNEALLNKAYVYAMQKHGSQKRASGDPYFSHPLEVAAILTDMH 60

Query: 160  LDEATIAIALLHDTIEDTTATRQEIDQLFGPEIGKLVEGLTKLKKLDLVSKKAVQAENLR 339
            LDEATIAIALLHDTIEDTTATRQEIDQLFGPEIGKLVEGLTKLKKLDLVSKKAVQAENLR
Sbjct: 61   LDEATIAIALLHDTIEDTTATRQEIDQLFGPEIGKLVEGLTKLKKLDLVSKKAVQAENLR 120

Query: 340  KLLLAISEDVRVLLVKLADRLHNMRTLGVMCEDKRLRIAEETMDIYAPLAGRMGMQDMRE 519
            KLLLAISEDVRVLLVKLADRLHNMRTLGVMCEDKRLRIAEETMDIYAPLAGRMGMQDMRE
Sbjct: 121  KLLLAISEDVRVLLVKLADRLHNMRTLGVMCEDKRLRIAEETMDIYAPLAGRMGMQDMRE 180

Query: 520  ELEELAFRYINPDAWRAVTDRLAELLEKNRGLLQKIETDLSEIFEKNGIKASVKSRQKKP 699
            ELEELAFRYINPDAWRAVTDRLAELLEKNRGLLQKIETDLSEIFEKNGIKASVKSRQKKP
Sbjct: 181  ELEELAFRYINPDAWRAVTDRLAELLEKNRGLLQKIETDLSEIFEKNGIKASVKSRQKKP 240

Query: 700  WSVFRKMETKGLSFEQLSDIFGFRVMVDTVQDCYRALGLIHTTWSMVPGRFKDYISTPKQ 879
            WSVFRKMETKGLSFEQLSDIFGFRVMVDTVQDCYRALGLIHTTWSMVPGRFKDYISTPKQ
Sbjct: 241  WSVFRKMETKGLSFEQLSDIFGFRVMVDTVQDCYRALGLIHTTWSMVPGRFKDYISTPKQ 300

Query: 880  NDYRSIHTTIIGPSRQRIELQIRTREMDEIAEFGVAAHSIYKDRGSANNPHKISTETNAY 1059
            NDYRSIHTTIIGPSRQRIELQIRTREMDEIAEFGVAAHSIYKDRGSANNPHKISTETNAY
Sbjct: 301  NDYRSIHTTIIGPSRQRIELQIRTREMDEIAEFGVAAHSIYKDRGSANNPHKISTETNAY 360

Query: 1060 AWLRQTIEQLSEGDNPEEFLEHTKLELFQDQVFCFTPKGRLIALPRGATPIDFAYAVHTD 1239
            AWLRQTIEQLSEGDNPEEFLEHTKLELFQDQVFCFTPKGRLIALPRGATPIDFAYAVHTD
Sbjct: 361  AWLRQTIEQLSEGDNPEEFLEHTKLELFQDQVFCFTPKGRLIALPRGATPIDFAYAVHTD 420

Query: 1240 IGDSCVGAKVNGRIMPLMTELKNGDEVDIIRSKAQVPPAAWESLVATGKARAAIRRATRS 1419
            IGDSCVGAKVNGRIMPLMTELKNGDEVDIIRSKAQVPPAAWESLVATGKARAAIRRATRS
Sbjct: 421  IGDSCVGAKVNGRIMPLMTELKNGDEVDIIRSKAQVPPAAWESLVATGKARAAIRRATRS 480
```

**The latter part of this record is omitted**

**Query= brucella-chr1_1847 # 1965361 # 1966137 # -1 # ID=1_1847;partial=00;start_type=ATG;rbs_motif=GGAGG;rbs_spacer=5-10bp;gc_cont=0.574**
**(777 letters)**

```
                                                                 Score    E
Sequences producing significant alignments:                      (bits)  Value

gb|CP006961.1|                                                     1100   0.0
gb|CP006962.1|                                                       31   1.0

>gb|CP006961.1|
          Length = 2107842

 Score = 1100 bits (769), Expect = 0.0
 Identities = 775/779 (99%), Gaps = 2/779 (0%)
 Strand = Plus / Plus
```

# The first part of this record is omitted

```
Query: 481     atgcgcaagatcgttgatgaaaaacgctcgcagattgccgatgcgcgtggcgcgggccgt 540
               ||||||||||||||||||||||||||||||||||||||||||||||||||||||||||||
Sbjct: 1024952 atgcgcaagatcgttgatgaaaaacgctcgcagattgccgatgcgcgtggcgcgggccgt 1025011


Query: 541     tttacggggcgcgacgcggaacctcgcgcggaaatgcgctcgggccatatgccgggtgcg 600
               ||||||||||||||||||||||||||||||||||||||||||||||||||||||||||||
Sbjct: 1025012 tttacggggcgcgacgcggaacctcgcgcggaaatgcgctcgggccatatgccgggtgcg 1025071


Query: 601     cgcaatgttcctgttacaaccctttccgaaaacggtgaattgaaagacctcgaaagcctg 660
               ||||||||||||||||||||||||||||||||||||||||||||||||||||||||||||
Sbjct: 1025072 cgcaatgttcctgttacaaccctttccgaaaacggtgaattgaaagacctcgaaagcctg 1025131


Query: 661     cgcaggatttttgacgaggcgggtatcgacctgtcggggccggtggtcaccagttgcggt 720
               ||||||||||||||||||||||||||||||||||||||||||||||||||||||||||||
Sbjct: 1025132 cgcaggatttttgacgaggcgggtatcgacctgtcggggccggtggtcaccagttgcggt 1025191


Query: 721     ttcggtgttaccgctgccgtgattacgctcgcgcttacctcg--ggggcaaaaggataa 777
               | ||||||||||||||||||||||||||||||||||||||||  |||||| ||||||||
Sbjct: 1025192 tccggtgttaccgctgccgtgattacgctcgcgcttacctcgctggggcacaaggataa 1025250
```

**Query= brucella-chr1_277 # 295318 # 296457 # -1 # ID=1_277;partial=00;start_type=ATG;rbs_motif=None;rbs_spacer=None;gc_cont=0.619**

**(1140 letters)**

```
                                                                  Score    E
Sequences producing significant alignments:                       (bits) Value

gb|CP006961.1|                                                       1625   0.0
gb|CP006962.1|                                                         35   0.079

>gb|CP006961.1|
          Length = 2107842

 Score = 1625 bits (1136), Expect = 0.0
 Identities = 1138/1140 (99%)
 Strand = Plus / Plus
```

# The first part of this record is omitted

```
Query: 781    ccttggcgcggccagcttgagaaaaccattgccatccttcgtgatcccgcgagcgccaaa 840
              ||||||||||||||||||||||||||||||||||||||||||||||||||||||||||||
Sbjct: 596520 ccttggcgcggccagcttgagaaaaccattgccatccttcgtgatcccgcgagcgccaaa 596579


Query: 841    caggcggaggcgaaaggtccgagcgccgaagatgtcgaagcggcttccacgctcagcgcc 900
              ||||||||||||||||||||||||||||||||||||||||||||||||||||||||||||
Sbjct: 596580 caggcggaggcgaaaggtccgagcgccgaagatgtcgaagcggcttccacgctcagcgcc 596639


Query: 901    agagatcggcaggcgatggtggaaggcatggttcaacgcctcgatgaaacacttcgccag 960
              ||||||||||||||||||||||||||||||||||||||||||||||||||||||||||||
Sbjct: 596640 agagatcggcaggcgatggtggaaggcatggttcaacgcctcgatgaaacacttcgccag 596699


Query: 961    aatggcggggatatcgatggctggaagcggctgggccgctcttatatgatcctcaaccgc 1020
              |||||||||||||||||||||||||||||||||| |||||| ||||||||||||||||||
Sbjct: 596700 aatggcggggatatcgatggctggaagcggctggtccgctcctatatgatcctcaaccgc 596759


Query: 1021   cgcaacgatgcgcaggatgcgctggctcgcggcatgaaggctcttcagggtgaaaaccgg 1080
              ||||||||||||||||||||||||||||||||||||||||||||||||||||||||||||
Sbjct: 596760 cgcaacgatgcgcaggatgcgctggctcgcggcatgaaggctcttcagggtgaaaaccgg 596819


Query: 1081   accgaacttcaaagcttcgccaccacacttggactggacgtggggactgcacaagaatga 1140
              ||||||||||||||||||||||||||||||||||||||||||||||||||||||||||||
Sbjct: 596820 accgaacttcaaagcttcgccaccacacttggactggacgtggggactgcacaagaatga 596879
```

**Query= brucella-chr1_995 # 1054415 # 1059196 # -1 # ID=1_995;partial=00;start_type=ATG;rbs_motif=GGAG/GAGG;rbs_spacer=5-10bp;gc_cont=0.522**
**(4782 letters)**

```
                                                                 Score    E
Sequences producing significant alignments:                      (bits)  Value

gb|CP006961.1|                                                     6823   0.0
gb|CP006962.1|                                                      242   2e-63

>gb|CP006961.1|
          Length = 2107842

 Score = 6823 bits (4776), Expect = 0.0
 Identities = 4780/4782 (99%), Gaps = 2/4782 (0%)
 Strand = Plus / Plus
```

# The first part of this record is omitted

```
Query: 3001    aaagctgatcttcatgattttaacacgtcagtcggggggggggggcttattggggcaggt 3060
               |||||||||||||||||||||||||||||||||  |||||||||||||||||||||||||
Sbjct: 1938311 aaagctgatcttcatgattttaacacgtcagtc--ggggggggggcttattggggcaggt 1938368


Query: 3061    gatgtaacgctcggcagcggaacattgactgtcaatcaaggcttcgacagtattttttcc 3120
               ||||||||||||||||||||||||||||||||||||||||||||||||||||||||||||
Sbjct: 1938369 gatgtaacgctcggcagcggaacattgactgtcaatcaaggcttcgacagtattttttcc 1938428


Query: 3121    ggcgtcatatctggagacggggggctgaacaaaagcggcgcggggcagttaactctttca 3180
               ||||||||||||||||||||||||||||||||||||||||||||||||||||||||||||
Sbjct: 1938429 ggcgtcatatctggagacggggggctgaacaaaagcggcgcggggcagttaactctttca 1938488


Query: 3181    ggcgccaatacttatggcggcgccacgaccattgatggaggggtgctccttcaaggtgag 3240
               ||||||||||||||||||||||||||||||||||||||||||||||||||||||||||||
Sbjct: 1938489 ggcgccaatacttatggcggcgccacgaccattgatggaggggtgctccttcaaggtgag 1938548


Query: 3241    agcggagctttcagcagtagttccgcctatcgaaccggcgcaagcggaaccgtggattta 3300
               ||||||||||||||||||||||||||||||||||||||||||||||||||||||||||||
Sbjct: 1938549 agcggagctttcagcagtagttccgcctatcgaaccggcgcaagcggaaccgtggattta 1938608
```

# The latter part of this record is omitted
